# The Importance of Language: A Quest for Accurate Definitions of Oromandibular Movement Disorders

**DOI:** 10.1002/mdc3.14119

**Published:** 2024-06-06

**Authors:** Merel C. Verhoeff, Frank Lobbezoo

**Affiliations:** ^1^ Department of Orofacial Pain and Dysfunction Academic Centre for Dentistry Amsterdam (ACTA), University of Amsterdam and Vrije Universiteit Amsterdam Amsterdam The Netherlands

**Keywords:** definition, oromandibular movement disorder, dyskinesia, dystonia, bruxism

Nelson Mandela once said: “If you talk to a man in a language he understands, that goes to his head. If you talk to him in his own language, that goes to his heart”. In other words: if we genuinely want to connect with each other, we have to speak each other's language. Translating this to healthcare, this means that we should promote interdisciplinary and interprofessional collaboration, and this starts with clear language, hence accurate definitions.

In otherwise healthy individuals, bruxism is defined as “a repetitive jaw‐muscle activity characterized by clenching or grinding of the teeth and/or by bracing or thrusting of the mandible”.^1^, ^2^ Bruxism can occur during sleep (indicated as sleep bruxism) or during wakefulness (indicated as awake bruxism)”. It can be a risk factor for negative health outcomes (eg, temporomandibular pain),^2^ but also a protective factor for positive health outcomes (eg, increasing upper airway patency).^2^ Therefore, bruxism is no longer considered a disorder but rather a motor behavior.^2^ However, in non‐healthy individuals, it is still unclear how to label the jaw‐muscle activities. Using “bruxism” for such activities is precluded by its current definition that is only valid in otherwise healthy individuals.

Traditionally, bruxism has been a major issue in dentistry, while medical doctors have primarily dealt with oromandibular movement disorders like dyskinesias, dystonia, and tremors. During an open peer‐review process, the reviewer asked a critical and empirical question: “For people with Parkinson's disease, is masticatory muscle activity ‘bruxism’ at all?”.^3^ Also Yoshida (2023) questioned whether oromandibular dystonia is bruxism or vice versa.^4^ Unfortunately, interprofessional collaboration on the issue of bruxism and oromandibular movement disorders has been hampered by a silo approach, with medicine and dentistry working in their own settings.^5^ Yet, similar characteristics can be found for bruxism and oromandibular movement disorders (eg, [partly] involuntary, recurrent, repetitive), although not every movement in the orofacial area is difficult to distinguish from bruxism (eg, jaw‐opening dystonia). The question therefore arises whether it is actually possible to distinguish bruxism and oromandibular movement disorders from one another by applying the existing definitions?

We hypothesize that there are three possible answers to this question: (a) there is no overlap between definitions, in which case the above‐identified silo approach could be acceptable after all; (b) there is partial overlap, suggesting that medical doctors and dentists should work together; and (c) the definitions are alike, so that we have to conclude that it is not correct that separate definitions exist. To examine these hypotheses, the bruxism definition should be expanded to include non‐healthy individuals; interdisciplinary expert meetings should be organized to identify all existing definitions of oromandibular movement disorders, to scrutinize their complexity, and to analyze the possible overlapping characteristics with bruxism; and research must be carried out, eg, with face‐recognition software, to address this issue from a clinical point of view.

After the completion of the above actions, it is possible to see if, and if so, to what extent, bruxism and oromandibular movement disorders overlap, and thus whether medical and dental professionals should collaborate more than they currently practice.

## Author Roles

(1) Research project: A. conceptualization; (2) Manuscript: A. writing, B. reviewing

M.C.V.: 1A, 2A

F.L.: 1A, 2B

## Disclosures

**Ethical Compliance Statement:** The authors confirm that the approval of an institutional review board, and patient consent was not required for this work. We confirm that we have read the Journal's position on issues involved in ethical publication and affirm that this work is consistent with those guidelines.

**Funding Sources and Conflict of Interest:** No specific funding was received for this work and the authors declare that there are no conflicts of interest relevant to this work.

**Financial Disclosures for the Previous 12 Months:** The authors declare that there are no additional disclosures to report.
